# Crossing the LINE Toward Genomic Instability: LINE-1 Retrotransposition in Cancer

**DOI:** 10.3389/fchem.2015.00068

**Published:** 2015-12-16

**Authors:** Jacqueline R. Kemp, Michelle S. Longworth

**Affiliations:** Department of Cellular and Molecular Medicine, Lerner Research Institute of Cleveland ClinicCleveland, OH, USA

**Keywords:** retrotransposons, LINE-1, genomic instability, retrotransposition, cancer

## Abstract

Retrotransposons are repetitive DNA sequences that are positioned throughout the human genome. Retrotransposons are capable of copying themselves and mobilizing new copies to novel genomic locations in a process called retrotransposition. While most retrotransposon sequences in the human genome are incomplete and incapable of mobilization, the LINE-1 retrotransposon, which comprises~17% of the human genome, remains active. The disruption of cellular mechanisms that suppress retrotransposon activity is linked to the generation of aneuploidy, a potential driver of tumor development. When retrotransposons insert into a novel genomic region, they have the potential to disrupt the coding sequence of endogenous genes and alter gene expression, which can lead to deleterious consequences for the organism. Additionally, increased LINE-1 copy numbers provide more chances for recombination events to occur between retrotransposons, which can lead to chromosomal breaks and rearrangements. LINE-1 activity is increased in various cancer cell lines and in patient tissues resected from primary tumors. LINE-1 activity also correlates with increased cancer metastasis. This review aims to give a brief overview of the connections between LINE-1 retrotransposition and the loss of genome stability. We will also discuss the mechanisms that repress retrotransposition in human cells and their links to cancer.

## The LINE-1 retrotransposon is an active mobile element

Retrotransposons, a class of transposable elements (TE), are highly repetitive DNA sequences positioned throughout the human genome. These structural elements make use of an RNA-mediated transposition process, allowing them to move from one location in the genome to another, while the original copy remains in its original locus. The RNA-based retrotransposons are classified into the autonomous long terminal repeat (LTR) and the non-LTR containing retrotransposons. LTR containing retrotransposons, as their name implies, possess LTRs ranging from 100 bp to over 5 kb in size and are endogenous retroviruses. Long interspersed nuclear elements (LINEs), comprising 20% of the human genome are a type of non-LTR retrotransposon. Non-autonomous retrotransposons are a third class of retrotransposons, of which the short interspersed nuclear elements (SINEs) comprise ~13% of the human genome (Lander et al., 2001).

The human genome contains millions of copies of retrotransposons; however, only a single non-LTR retrotransposon family, the LINE-1 (L1) family, remains the primary source of retrotransposition. The activity of the L1 retrotransposon has persisted over time within the human genome and its de-repression is associated with genomic instability and tumor development (Gasior et al., 2006; Lee et al., 2012). Over 100,000 L1 sequences exist in the human genome; however, most are rendered inactive by point mutations, rearrangements, or truncations (Brouha et al., 2003). It was originally estimated that the average human diploid genome contains ~80–100 active L1s that are capable of undergoing retrotransposition (Sassaman et al., 1997). Of those which are active, six were classified as “hot” L1s responsible for the bulk of L1 retrotransposition within the human genome (Brouha et al., 2003). More recently, however, three independent studies demonstrated that the occurrence of new L1 insertions is more prevalent than previously thought. Additionally, a number of the newly inserted “hot” L1s were found to be extremely polymorphic and specific to a few individuals, suggesting that L1 retrotransposition may contribute to the propensity for one individual to develop disease over another (Beck et al., 2010; Huang et al., 2010; Iskow et al., 2010).

A full-length L1 retrotransposon is ~6 kB in size and contains a 5′ untranslated region, two non-overlapping open reading frames (ORF1 and ORF2), and a 3′ untranslated region that ends in a poly (A) tail (Swergold, 1990; Becker et al., 1993). ORF1 encodes a 40 kDa RNA-binding protein (Mathias et al., 1991), whereas ORF2 encodes a 150 kDa protein (ORF2p) with demonstrated endonuclease and reverse transcriptase activities (Mathias et al., 1991; Feng et al., 1996; Piskareva et al., 2003). Interestingly, ORF2p also contains a conserved cysteine-rich domain recently shown to have a high non-specific affinity to RNA, which may contribute to the process of reverse transcription (Piskareva et al., 2013). Various mutants of ORF1p and ORF2p, have been created and used to demonstrate that the two proteins are necessary for retrotransposition in a cell culture based assay (Moran et al., 1996; Wei et al., 2001; Kulpa and Moran, 2005; Doucet et al., 2010).

**Figure 1:**
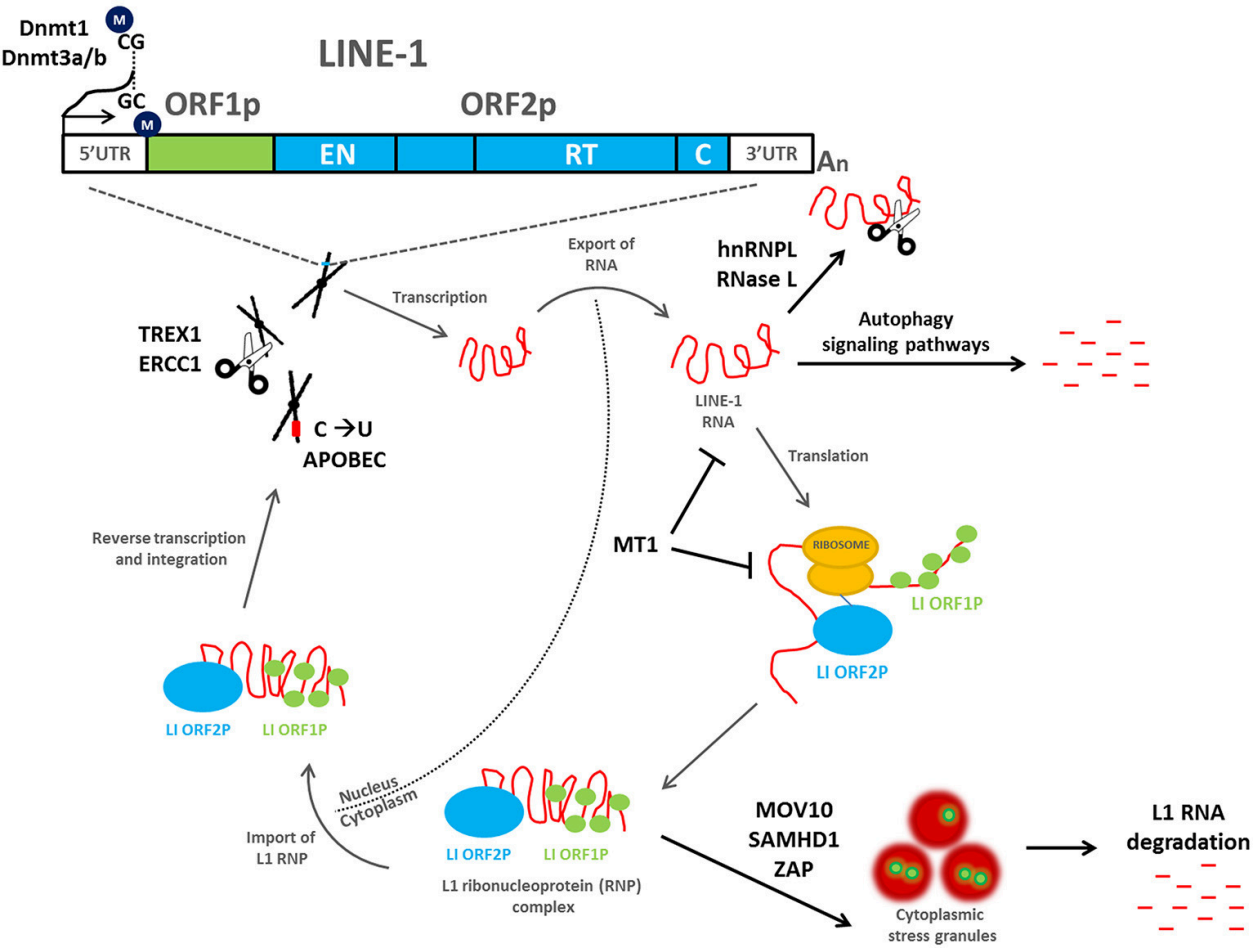
**Mobilization of L1 retrotransposons and the cellular mechanisms that inhibit their retrotransposition**. A full-length L1 retrotransposon contains a 5′ untranslated region, two non-overlapping open reading frames (ORF1 and ORF2), and a 3′ untranslated region that ends in a poly (A) tail. ORF1 encodes a 40 kDa RNA-binding protein, whereas ORF2 encodes a 150 kDa protein (ORF2p) with demonstrated endonuclease (EN) and reverse transcriptase (RT) activities. During a cycle of retrotransposition (gray arrows), L1 is transcribed and exported into the cytoplasm, where translation occurs. ORF1p and ORF2p preferentially bind to their own mRNA and form ribonucleoprotein (RNP) complexes. The L1 RNP gains access into the nucleus, where the ORF2p endonuclease domain cleaves genomic DNA to expose a 3′-hydroxyl residue that is used as a primer by the L1 reverse transcriptase to copy the L1 mRNA, a mechanism that has been termed target-primed reverse transcription (TPRT). The resulting cDNA is then inserted into a novel region in the genome. A number of host cell defense mechanisms exist to inhibit L1 retrotransposition (black arrows), including L1 DNA methylation, mutation, and/or degradation, L1 RNA degradation, inhibition of L1 RNP formation, and/or localization to stress granules, and autophagy signaling pathways. All are capable of inhibiting L1 and preventing its mobilization throughout the human genome.

The mobility of a L1 retrotransposon is completely dependent on transcription and translation of its encoded proteins and therefore incudes both nuclear and cytoplasmic events essential for retrotransposon duplication (Figure 1). ORF1p and ORF2p preferentially bind to their own mRNA and form ribonucleoprotein (RNP) complexes (Leibold et al., 1990; Alisch et al., 2006; Dmitriev et al., 2007; Doucet et al., 2010). ORF1p has been demonstrated to have nucleic acid chaperone activity that is essential for the retrotransposition process (Martin et al., 2005, 2008). The L1 RNP gains access into the nucleus, where the ORF2p endonuclease domain cleaves genomic DNA to expose a 3′-hydroxyl residue that is used as a primer by the L1 reverse transcriptase to copy the L1 mRNA, a mechanism that has been termed target-primed reverse transcription (TPRT). The resulting cDNA is then inserted into a novel region in the genome (Cost et al., 2002). A nuclear localization signal has been identified in ORF2p (Goodier et al., 2004); however, it is unclear whether the L1 RNP is capable of crossing an intact nuclear membrane or whether it gains access following nuclear envelope breakdown (Kubo et al., 2006).

## Potential L1-mediated mechanisms of tumor development

Many reports have demonstrated that retrotransposons can significantly impact the structure of the human genome. Retrotransposons have adverse effects on genome stability since multiple copies of the same sequence can hinder precise chromosomal pairing during mitosis and meiosis, resulting in DNA double-stranded breaks, more homologous recombination, chromosome duplication, and increased potential for inefficient repair of recombination events (Belgnaoui et al., 2006; Farkash et al., 2006; Gasior et al., 2006). A recent study identified LINE-LINE-mediated non-allelic homologous recombination as an important mechanism of structural rearrangement, contributing to genomic variability and instability (Startek et al., 2015).

L1 retrotransposition events in the human genome have been deemed responsible for ~97 disease-producing insertions (reviewed in Hancks and Kazazian, 2012). Specifically, direct insertional mutagenesis caused by L1 retrotransposition can result in disruption of coding sequence, disruption of splicing, and/or deregulation of gene expression. Symer and colleagues identified L1 element inversions, extra nucleotide insertions, exon deletions, a chromosomal inversion, and flanking sequence comobilization in the retrotransposon target site in human tissue culture cells (Symer et al., 2002). Studies have also shown that L1 acts as more than just an insertional mutagen, but also that its retrotransposition activity can result in large genomic deletions (Gilbert et al., 2002).

L1 retrotransposons exhibit a *cis*-preference, in which the L1 proteins preferentially use their own L1 RNA as the transcript for reverse transcription and integration (Wei et al., 2001; Kulpa and Moran, 2006). However, L1 proteins can also work in *trans* to promote mobilization of other RNAs, thus increasing their potential for causing genomic instability. Non-autonomous elements including SINEs (Dewannieux et al., 2003) and SVAs (Raiz et al., 2012), as well as small nuclear RNAs (e.g., U6 snRNA; Buzdin et al., 2002; Gilbert et al., 2005; Garcia-Perez et al., 2007), small nucleolar RNAs (e.g., U3 snoRNA; Weber, 2006), and messenger RNAs (Esnault et al., 2000; Wei et al., 2001) are all capable of being *trans*-mobilized via L1. In all of these cases, retrotransposition of mRNAs results in processed pseudogenes that bear L1 structural hallmarks. These *trans*-mobilization events utilize the ORF1p and/or ORF2p to insert into the human genome and do not involve sequence specificity. Once these pseudogenes are inserted back into the genome, they usually lack introns and promoters, but contain a poly (A) 3′ end and target-site duplications of varying length (Vanin, 1985; Weiner et al., 1986; Esnault et al., 2000). Interestingly, siRNAs have been shown to be expressed from pseudogenes in mouse oocytes, suggesting a potential way in which they might influence gene regulation (Tam et al., 2008). Therefore, generation of processed pseudogenes is a direct product of endogenous retrotransposition activity in the human genome that can contribute to genomic diversity and instability.

Integration of L1 in or near oncogenes or tumor suppressor genes can contribute to tumor development (Morse et al., 1988; Miki et al., 1992; Iskow et al., 2010) and progression of life-threatening cancers, including lung, colon, and breast cancer in humans (Lee et al., 2012; Criscione et al., 2014). For example, disruption of the APC gene by a somatic insertion of L1 was shown to be present in colon cancer and associated with development of colorectal tumors (Miki et al., 1992). The APC gene encodes a tumor suppressor involved in maintaining chromosomal stability during mitosis (Fodde et al., 2001b). In *Apc* deficient mouse cells, structural rearrangements, resulting from chromosomal breakage and recombination are apparent (Fodde et al., 2001a). Further, cells are defective in chromosome segregation when they carry a truncated form of *Apc* (Kaplan et al., 2001). Other tumor suppressor genes found to be disrupted by tumor-specific L1 insertions include Mutated in Colorectal Cancers (MCC) and Suppression of Tumorigenicity 18 (ST18; Shukla et al., 2013). Furthermore, since L1 machinery acts to *trans*-mobilize other RNAs, those insertions can also impact expression of genes. *Alu*, a type of SINE present in higher copy numbers than L1, can be *trans-*mobilized, leading to cancer-associated gene insertions. Sites of *Alu* insertions include the APC locus and this was associated with Desmoids tumors (Halling et al., 1999), the tumor suppressor NF-1 (neurofibromatosis type I; Wallace et al., 1991), and the BRCA1 and BRCA2 breast/ovarian cancer related genes (Miki et al., 1996; Teugels et al., 2005). SVA elements can also be mobilized by the L1 retrotransposition machinery, leading to disease (Ostertag et al., 2003). In one study, mobilization of SVA resulted in deletion of the *HLA-A* gene in three Japanese families; a number of individuals in these families were afflicted with leukemia (Takasu et al., 2007).

Telomerase reactivation, as a means to maintain telomeres, occurs in the early stages of carcinogenesis to promote cancer cell immortalization (Counter et al., 1992; Kim et al., 1994). Transcriptional regulation of hTERT, the catalytic subunit of telomerase, is a major mechanism for telomerase activation in the cancer setting. In a recent study, L1 was shown to contribute to tumor pathogenicity by inducing hTERT and helping to maintain telomeres in telomerase-positive tumor cells. Depletion of L1 resulted in reduced telomere length, suggesting that L1 is a reasonable target in the treatment of telomerase-positive cancer (Aschacher et al., 2015).

## L1 expression in cancers

Given that L1 retrotransposition can lead to genomic instability and genetic heterogeneity is a common feature in tumor initiating cells, it is not surprising that expression of the L1-encoded ORF1p is reported to be a hallmark of many human cancers, with almost half (47%) of the human neoplasms examined being immunoreactive for L1 (Rodic et al., 2014). L1 positive neoplasms included invasive breast carcinomas (97% L1 positive), high-grade ovarian carcinomas (91.5% L1 positive), and pancreatic ductal adenocarcinomas (PDACs; 89% L1 positive). Carcinomas originating in the endometrium, biliary tract, esophagus, bladder, head and neck, lung, and colon were also frequently L1 immunoreactive (22.6–76.7% L1 positive; Rodic et al., 2014). In a separate study, increased ORF1p expression and novel L1 insertions in PDAC were observed in matched primary and metastatic tissues. However, the overall results showed discordant rates of retrotransposition, suggesting that while increased L1 retrotransposition may not be a direct cause of metastatic PDAC, it may contribute to gene disregulation leading to metastasis (Rodic et al., 2015). Furthermore, activation of L1 increases the risk of epithelial-mesenchymal transition and metastasis in epithelial cancer (reviewed in Rangasamy et al., 2015) and promotes proliferation and invasion of LoVo colorectal cancer cells (Li et al., 2014) and MDA-MB-231 breast cancer cells (Yang et al., 2013). ORF1p and ORF2p levels are upregulated in breast cancers compared to normal tissues. Cytoplasmic levels of ORF1p and ORF2p are elevated in DCIS breast cancers compared to highly invasive cancers. Conversely, nuclear levels of ORF1p and ORF2p were found to be higher in invasive breast cancers and correlated with increased lymph node metastasis and poor patient survival (Harris et al., 2010; Chen et al., 2012). Furthermore, inhibition of the L1-encoded reverse transcriptase in breast cancer cells was demonstrated to reduce the rate of proliferation and promote cellular differentiation (Patnala et al., 2014). Finally, L1 activity and expression was elevated in rat chloroleukemia cells, suggesting that mobilization of this retrotransposon may contribute to the genomic instability observed in this model of blood cancer (Kirilyuk et al., 2008).

Hypomethylation of L1 DNA has been observed in various cancers and is associated with an increase in transcriptional activation and expression of L1 (Alves et al., 1996; Asch et al., 1996; Kitkumthorn et al., 2012; Murata et al., 2013; Criscione et al., 2014; Park et al., 2014). L1 hypomethylation can occur early in tumorigenesis and is associated with bladder (Patchsung et al., 2012; Salas et al., 2014), gastric (Shigaki et al., 2013; Baba et al., 2014a), colon (Ogino et al., 2008; Antelo et al., 2012; Murata et al., 2013), lung (Saito et al., 2010), and breast cancers (Park et al., 2014). L1 hypomethylation is associated with poor prognosis of lung adenocarcinoma (Ikeda et al., 2013), hepatocellular carcinoma via activation of c-Met (Zhu et al., 2014), esophageal squamous cell carcinoma (ESCC; Iwagami et al., 2013), and with inferior survival in colorectal carcinomas with high microsatellite instability (Inamura et al., 2014). Additionally, L1 hypomethylation in ESCC was shown to be significantly associated with lymph node metastasis, frequency of p53 mutation, and chromosomal instability (Kawano et al., 2014). In a separate study, L1 hypomethylation in ESCC patient samples was associated with an increase in CDK6 expression (Baba et al., 2014b). This may contribute to the aggressiveness of tumors since CDK6 is known to promote tumor progression by stimulating proliferation and angiogenesis (Kollmann et al., 2013). Finally, hypomethylation of L1 in colorectal cancer can lead to activation of oncogenes important in metastasis, including MET, RAB1P, and CHRM3 (Hur et al., 2014). It was observed that specific L1 sequences residing within the intronic regions of these proto-oncogenes were hypomethylated and reduced methylation of specific L1 elements within the MET gene correlated with an induction of MET expression (Hur et al., 2014). However, since methylation levels of repetitive L1 elements often tightly correlate with global DNA methylation levels, it is difficult to conclude that L1 hypomethylation directly results in the increased genomic instability found in tumors.

## Mechanisms that inhibit L1 retrotransposition are often deregulated in cancer

As the uncontrolled movement of retrotransposons throughout the genome can have deleterious consequences for genome stability and health in general, a number of defense mechanisms exist in human cells to repress their movement. These mechanisms exist at the DNA, RNA, and protein levels to inhibit L1 and retrotransposition (Figure 1).

DNA methylation status is a major determinant of gene expression changes within the human genome and is involved in various biological processes including cancer (Liu et al., 2003). As discussed above, hypomethylation of L1 DNA is associated with an increase in L1 expression. Conversely, methylation of L1 within the CpG rich 5′-UTR represses its ability to be activated and transcribed, thereby minimizing the exposure of genomic DNA to L1-associated damage (Hata and Sakaki, 1997; Weisenberger et al., 2005; Barchitta et al., 2014). DNA methylation, therefore, is a key mechanism for L1 silencing. It has been shown in mouse embryonic stem cells that methylation of the L1 promoter is maintained by DNA methylatransferases, including Dmnt1 and Dmnt3a and/or -3b (Woodcock et al., 1998; Liang et al., 2002).

Other epigenetic mechanisms have been reported to be involved in regulating L1 expression. One study showed that reporter genes introduced into human embryonic carcinoma-derived cell lines by engineered L1 retrotransposons were rapidly silenced during or shortly after their integration (Garcia-Perez et al., 2010). Treatment of the cells with histone deacetylase inhibitors reversed the silencing and ChIP experiments demonstrated that a change in the chromatin status at the L1 integration site correlated with reactivation of the reporter gene (Garcia-Perez et al., 2010). Other studies involving chromatin structure averaged global histone modifications and found that histone H3 lysine nine methylation is enriched at human retrotransposons, suggesting that histone methylation may play a role in repressing recombination of these retrotransposons (Kondo and Issa, 2003; Martens et al., 2005; Goodier and Kazazian, 2008). Low levels of the silencing histone modification H3K27me3 at L1 loci in conjunction with L1 hypomethylation has been shown to support an active role in rectal cancer prognosis and a poor clinical outcome (Benard et al., 2013). Conversely, high levels of the activating histone modification H3K9Ac at L1 loci were associated with poor patient survival. This indicates that L1 methylation and histone modifications work closely together in determining gene expression and tumor progression (Benard et al., 2013).

Global chromatin organization is also involved in repression of *Drosophila melanogaster* retrotransposons. Studies in the fly have identified a role for the chromatin organizing complex, Condensin II in repressing retrotransposition in somatic cells and tissues. The Condensin II subunit, dCAP-D3, promotes silencing of retrotransposon-containing loci by maintaining boundaries of repressive histone modifications to repress retrotransposon transcription and ultimately inhibit retrotransposition (Schuster et al., 2013). Furthermore, decreased dCAP-D3 expression impacts chromatin structure, resulting in DNA double strand breaks within the retrotransposon sequence, an increase in homologous pairing, and an increase in global retrotransposon copy number. While global chromatin regulators have yet to be implemented in L1 repression, CAP-D3, and Condensin II are conserved and further studies are necessary to determine whether they also inhibit retrotransposition in human cells.

Epigenetic modification, however, is not the only mechanism employed by cells to inhibit retrotransposition. Exciting new evidence from multiple labs suggests that a host of cellular proteins employ distinct mechanisms to accomplish the inhibition.

One mechanism includes targeting the L1 RNA intermediate to prohibit insertion of L1 into the human genome. The ribonucleoprotein hnRNPL, which plays multiple roles in RNA metabolism, has been shown to directly interact with L1 RNA to negatively regulate retrotransposition. hnRNPL does so by decreasing the steady-state levels of the L1 RNA (Peddigari et al., 2013). Downregulation of L1 mRNA and subsequently, reduced expression of the ORF1p and ORF2p by RNase L was also shown to restrict L1 mobilization; whereas, siRNA-mediated knockdown of endogenous RNase L lead to a significant increase in L1 retrotransposition events in a human ovarian cancer cell line (Zhang et al., 2014). Similarly, the melatonin receptor 1 (MT1) inhibits retrotransposition through downregulation of L1 mRNA and ORF1p. Researchers showed that antagonists directed against MT1 abolished this effect in a dose-dependent manner (deHaro et al., 2014). Furthermore, melatonin-rich blood suppressed endogenous L1 RNA during *in situ* perfusion of tissue-isolated xenografts of human pancreatic cancer (deHaro et al., 2014).

Innate immune defenses can also inhibit retrotransposition of L1. Guo and colleagues demonstrated that autophagy degrades the L1 RNA intermediate, preventing new insertions into the genome and promoting genome stability. Degradation of retrotransposon RNA was facilitated by receptors involved in activating autophagy signaling pathways, NDP52 and p62. Interestingly, this study also showed that mice lacking *Atg6/Beclin1*, a gene critical for the formation of autophagosomes, accumulate retrotransposon RNA and new genomic insertions of L1 (Guo et al., 2014).

L1 RNP formation and safe delivery of the RNP to genomic DNA is essential for TPRT to occur; therefore, targeting the RNP for degradation is a useful mechanism to inhibit this process. The RNA helicase, MOV10 directly associates with the L1 RNP (Goodier et al., 2012) and similar to SAMHD1 (Zhao et al., 2013) inhibits L1 retrotransposition by promoting stress granule formation (Arjan-Odedra et al., 2012; Li et al., 2013); stress granules are ribonucleoprotein cytosolic foci that appear under cellular stress and often act to promote mRNA degradation (Kedersha et al., 2005). Further, the L1 ORF1p was shown in a separate study to localize in stress granules with components of RISC, suggesting a mechanism for controlling retrotransposition and the associated genomic damage (Goodier et al., 2007). More recently, the zinc-finger antiviral protein, ZAP was shown to inhibit L1 retrotransposition by binding to the L1 RNP and inhibiting accumulation of L1 RNA (Goodier et al., 2015; Moldovan and Moran, 2015). ZAP colocalizes with the RNP in cytoplasmic stress granules and interacts with a number of novel proteins, including MOV10 (Goodier et al., 2015; Moldovan and Moran, 2015).

Another mechanism to inhibit retrotransposition involves targeting the single-strand DNA that arises during the process of L1 integration, to repress its mobilization. These cellular proteins can directly promote degradation of L1, thereby inhibiting retrotransposition. For example, the APOBEC3 (A3) family of cytidine deaminases functions to inhibit L1 retrotransposition by deaminating the transiently exposed cDNA, creating C-to-U conversions (Richardson et al., 2014). This may then target the mutated retrotransposon DNA for degradation through endonuclease activity. Additionally, the endonucleases TREX1 (Stetson et al., 2008) and ERCC1/XPF (Gasior et al., 2008) can physically cleave the reverse-transcribed cDNA of L1, thereby inhibiting retrotransposition.

## Conclusions

Undeniably, L1 retrotransposons are an interesting and important component of the human genome. The activity of L1 retrotransposons can generate a wide array of genomic mutations and rearrangements, with potentially serious consequences for the stability of the genome. L1s are frequently hypomethylated and expressed in human cancers and their increased activity correlates with tumor progression and metastasis. Additionally, L1-insertion-mediated interference with normal RNA processing and expression also contributes to cancer development. Further studies on L1 retrotransposition, their effects on local and global genome organization, and the identification of novel mechanisms which repress retrotransposition to prevent tumor development will broaden our understanding of the impact of retrotransposons on genetic diversity and human health.

## Funding

This work was supported by a National Institutes of Health research grant R01GM102400 to ML. The contents are solely the responsibility of the authors and do not necessarily represent the official views of the NIH.

### Conflict of interest statement

The authors declare that the research was conducted in the absence of any commercial or financial relationships that could be construed as a potential conflict of interest.
